# Obstetrics and Gynecology

**Published:** 1894-07

**Authors:** 


### *SELECTIONS AND ABSTRACTS.*

---

### OBSTETRICS AND GYNECOLOGY.

Edited by Dr. VIRGIL O. HARDON.

### A New Indication for Supravaginal Hysterectomy.

Under this title, Lauro (*Rif. Med.*, October 23d and 24th, 1893) describes a case occurring in his own practice, and takes the opportunity of reviewing the indications for operative interference in displacements of the uterus. His conclusions are as follows: (1) In sexually active women, affected with retrodeviation of the uterus without any adhesions to the walls of the pelvis posteriorly, the intense sufferings in such cases can often be relieved by Dr. Alexander's operation, the severer operation of hysterectomy being thus unnecessary. (2) During reproductive life, in a woman afflicted with retroflexion or retroversion complicated by adhesions, the organ should be freed from its adhesions, and the round ligaments shortened intraperitoneally. This gives better results than ventrofixation of the organ. (3) In case of failure of these measures, recourse should be had to hysteropexy, by which means the organ can be more solidly fixed, without in general interfering with normal involution in future gestations. (4) Supposing laparotomy to have failed to prevent the return of the retrodeviation, and life to be in consequence a burden to the patient, one is then justified in suggesting extirpation of the reproductive organs. But this should never be done without a previous consultation. (5) In such cases the operation to be preferred is an abdominal hysterectomy, so that adhesions contracted as a result perhaps of former operations, with the abdominal organs may be better dealt with. Such adhesions are often missed even by the most careful examiner before the operation. (6) If the menopause is past, there need be less hesitation in proceeding to hysterectomy. (7) The two operations, abdominal and vaginal hysterectomy, seem to differ but little on the score of danger to the patient, as in both cases the peritoneal sac has to be opened.

---

### TREATMENT OF CHRONIC CERVICAL ENDOMETRITIS IN NULLIPARA.

At a meeting in February, 1893, of the Surgical Society of Paris, Dr. Bouilly spoke on this subject. The author wished that it be understood that he only was speaking of chronic endometritis as met with in young women who had never had children, and, consequently, had neither tear nor sclero-hystic degeneration of the uterus. In these subjects the cavity of the cervix is narrow, and the secretions are retained. Women who have sticky, viscous discharges are sterile, but suffer little, having only a feeling of weight in the pelvis. The author thought that perhaps gonorrhea was the cause. As curetting the uterus and topical applications always fail, and as Schroeder's operation is too complicated for so small a lesion, the author proposes the following operation: He commences by dilating the uterus for two days, after which he washes out the cavity and curettes the uterine cavity, the upper and lower lip of the organ are fixed by forceps, and the operation proper is commenced. With a long narrow-bladed bistoury a flap two, three, or four millimetres thick, according to the thickness of the tissue, is removed from the demi-circumference of each lip. The mucus is thus abraded so as to form two excavations with their cavity opposed to each other; the mucus on the sides of the cervix is carefully preserved, as it is necessary for the regeneration of this tissue. The external orifice is widely opened, and the cavity of the cervix is stuffed with iodoform gauze. The author has performed this operation forty times with only one failure, and twice conception has taken place in women who had been sterile for a long time. As little hemorrhages have taken place after operation was completed, the packing with the gauze should be carefully done—*Annals of Gynecology.*

### LIGATION OF THE BASE OF THE BROAD LIGAMENTS PER VAGINAM, INCLUDING THE UTERINE ARTERIES FOR FIBROIDS OF THE UTERUS.

Dr. Augustin H. Goelet, of New York, in a contribution to the *American Medico-Surgical Bulletin*, June 1st, reports favorably upon this operation in his hands for the control of uterine hemorrhage and reduction of fibroid growths. He believes it should be done in lieu of hysterectomy when that operation would involve too great a risk, and as a preliminary step with a view of avoiding the necessity of the more hazardous operation. When extensive attachments have not been formed which would afford additional nutrition considerable reduction has resulted even in growths of large size. When the operation has been done for smaller growths the result has been more satisfactory. In some instances complete atrophy has been reported. This result, as well as the arrest of the uterine hemorrhage, is accounted for by the diminished nutrition furnished the uterus and these growths by interference with the blood supply and nerve supply which is included by ligation of the base of the broad ligaments. It is estimated that the uterine arteries furnish the uterus with two-thirds of its blood supply, and it is reasonable to expect that a profound effect will be produced upon that organ and growths arising from the walls if this is suddenly cut off.

The sole danger in the operation is the risk of including the ureters in the ligatures, as they pass down behind the uterine arteries only a half an inch from the cervix, and are consequently in the field of operation. Dr. Goelet suggests as a preliminary step, to eliminate this risk, that bougies be passed into the ureters through the bladder. He admits, however, that a careful operator accustomed to working in this region may easily avoid the ureters.

The technique of the operation, as described by Dr. Goelet, shows an important departure from the usual method followed. Instead of ligating each artery in only one place on a level with the internal os, he applies a second and often a third ligature to the artery on each side as it ascends along the side of the uterus, the result of which is to cut off the compensating blood supply from ovarian artery to the lower part of the uterus.

Dr. Goelet gives all the credit of priority to Dr. Martin, of Chicago, who has recently suggested and popularized the operation and perfected its technique, but states that he first ligated the uterine artery *per vaginam* on one side in January, 1889, in the case of a large fibroid the size of a seven months' pregnancy, with a view of diminishing the size of the growth by reducing the blood supply. The artery on the other side was not ligated because the position of the tumor made it inaccessible. Six months later the tumor was one-third smaller, and was giving no inconvenience.

He quoted his last case operated upon to show how promptly uterine hemorrhage may be controlled by this operation.

---

### The Drainage of Pyosalpinx through the Uterus.

In a paper read before the American Gynecological Society, Dr. Murray joins with Dr. Polk and others in claiming that pyosalpinx can be cured without resort to the removal of the offending organ. The patient must be anesthetized, the uterus dilated and then thoroughly curetted. He formulates the following conclusions:

1. That many cases of pyosalpinx are curable without mutilating operations if the endometritis be treated by curettage and drainage with strict antiseptic percautions.

2. That uterine curettage and drainage should be practiced in every case before operation unless the tubes are very distended and thin, to cure the endometritis which may be and often is cause of trouble and of the lack of relief after celiotomy and removal of organs have been performed.

3. That even after pyosalpinx frequently the tubes and ovaries are not useless organs, the proof being that pregnancy occurs and the puerperium is normal.

4. That only after proper treatment, the tubes, ovaries and uterus still remaining bound down by adhesions and continuing a menace to life and health, should the radical operation be done.—*Pacific Medical Journal.*

